# Decoding the spread of non-indigenous fishes in the Mediterranean Sea

**DOI:** 10.1038/s41598-024-57109-8

**Published:** 2024-03-20

**Authors:** Georgios Vagenas, Paraskevi K. Karachle, Anthi Oikonomou, Maria Th. Stoumboudi, Argyro Zenetos

**Affiliations:** Institute of Marine Biological Resources and Inland Waters, Hellenic Centre for Marine Research, 46.7km Athens-Sounio Av., 19013 Anavissos, Greece

**Keywords:** Ecology, Climate-change ecology, Invasive species

## Abstract

The ocean is dynamically changing due to the influence of climate processes and human activities. The construction of the Suez Canal in the late nineteenth century opened the Pandora’s box by facilitating the dispersal of Red Sea species in the Mediterranean Sea. In this study, we developed an open-source spatio-temporal numerical analysis framework to decodify the complex spread of Mediterranean non-indigenous fish species (NIS) that entered through the Suez Canal. We utilized 772 historical detection records of 130 NIS to disentangle their dynamic spread through space and time. The results indicated that species follow a north-westward trajectory with an average expansion time step of 2.5 years. Additionally, we estimated the overall time for a NIS to reach the Central Mediterranean Sea from the Suez Canal at approximately 22 years. Based on the analysis, more than half of the introduced fishes have been established in less than 10 years. Finally, we proceeded in the cross-validation of our results using actual spread patterns of invasive fishes of the Mediterranean Sea, resulting up to 90% of temporal and spatial agreement. The methodology and the findings presented herein may contribute to management initiatives in highly invaded regions around the globe.

## Introduction

A robust consensus exists in the scientific world around the globe on the fact that climate change is mainly related to human activities^1^. Among the severe impacts that human interventions pose to the phenomenon of life on Earth^2^, warmer climate regimes influence the distribution of species towards a polar shift movement^3^. An adjunctive factor leading to species’ movement in multiple aquatic areas that have not been historically recorded concern human activities related to aquaculture, shipping, corridors, transportation and aquarium trade^4^. In this context, the onset of non-indigenous species (NIS) has emerged as a serious ecological threat that leads to significant habitat alterations, distributional shifts and extirpations of aquatic species along with quantitative and qualitative losses of ecosystem services^5,6^.

It has been more than a decade ago when the United Nations (UN) Convention of Biological Diversity established a 10-year Strategic Plan for Biodiversity. The Conservation Strategic Plan included, amongst others, the Aichi Target 9 which postulated that by 2020 the invasive alien species and pathways are identified and prioritized, the priority species are controlled or eradicated and that measures are enforced to manage pathways and prevent the introduction and establishment of invasive species^7^. However, until the end of 2020, the UN reported that the Aichi Target 9 has been “*partially achieved*”, since there was no evidence of upholding the number of new invasive species’ introductions^8^. A recent dystopic example is that, during the Aichi Targets’ decadal implementation period, the Mediterranean Sea has faced an increase of 40%, regarding marine invasive species’ establishment, while more than 1000 NIS have been recorded in the Mediterranean up to date^9,10^.

The Mediterranean Sea is one of the most invaded marine ecosystems in the world ^11,12^, whereas non-indigenous species’ introductions occur either through accidental or intentional human-induced vectors, namely transport corridors, aquaria releases, aquaculture escapes or stowaway^13^. The opening of the Suez Canal in 1869 is described as the main factor that led to the unintentional dispersal of NIS in the Eastern Mediterranean^14^. In conjunction with the increasing temperature in the Eastern Mediterranean basin^15^, the subsequent technical infrastructures in the region (i.e., dissolution of salt beds, construction of Aswan Dam, Suez Canal expansion^15,16^), have facilitated environmental changes that resulted in suitable habitat conditions for the Red Sea species, and thus their respective introduction and establishment. As one would expect, the so called “*Lessepsian migration*”^17^ has attracted the interest of the scientific community, since NIS pose serious ecological and socio-economic impacts^6,18^. For example, it has been reported that the gross estimation of marine invasions’ costs in the Mediterranean has risen up to 30 billion USD over the last three decades^19^.

In the Mediterranean, interdisciplinary scientific initiatives have attempted to disentangle the phenomenon of marine invasions either through the ecological investigation of species’ dispersal and the responses of NIS in native communities^20^, the integration of species’ traits to interpret NIS successive expansion^21,22^, or by employing a time series approach to explore the rate of introductions^23^. In the last decade, an additional novel approach has emerged by using advanced ecological models to project the distribution of NIS in the near future (i.e., Species Distribution Models—SDMs) based on predicted environmental thresholds^24,25^. Even though their application is of paramount importance in marine management planning, SDMs in marine environments have been perceived with caution for being stationary or niche conservatism^26^, while it is known that invasive species’ expansion is also controlled by the synergistic effect of non-climatic factors (e.g., habitat characteristics, human interventions) and multispecies interactions^27^. For these reasons, previous notable research focused on the dynamic spread of NIS by encompassing trends of historical introduction records^28^ and by taking into account time lags of detection, arrival and establishment^15,29^. However, large-scale research efforts related to the successive movement analysis of NIS in various spatial and temporal scales are rather limited in the Mediterranean Sea.

Herein, we analyze the dynamic spread of all NIS fishes that entered through the Suez Canal in the Mediterranean Sea over the last 141 years (i.e., 1882–2023). We propose a unified spatial and temporal numerical analysis framework based on published NIS detection records, in order to unravel hotspots of NIS introductions and to investigate dynamic time intervals across consecutive sightings. For this purpose, we explored the dominant hotspots of NIS in the Mediterranean Sea through a momentum analysis approach. We examined the main hypothesis that a common trajectory exists in the distributional expansion of NIS, or with simpler words, that NIS follow the same trajectory in their expansion in the Mediterranean. If this is not the case, multispecies complexity does not allow for the generalization of invading patterns.

## Results

**Figure 1:**
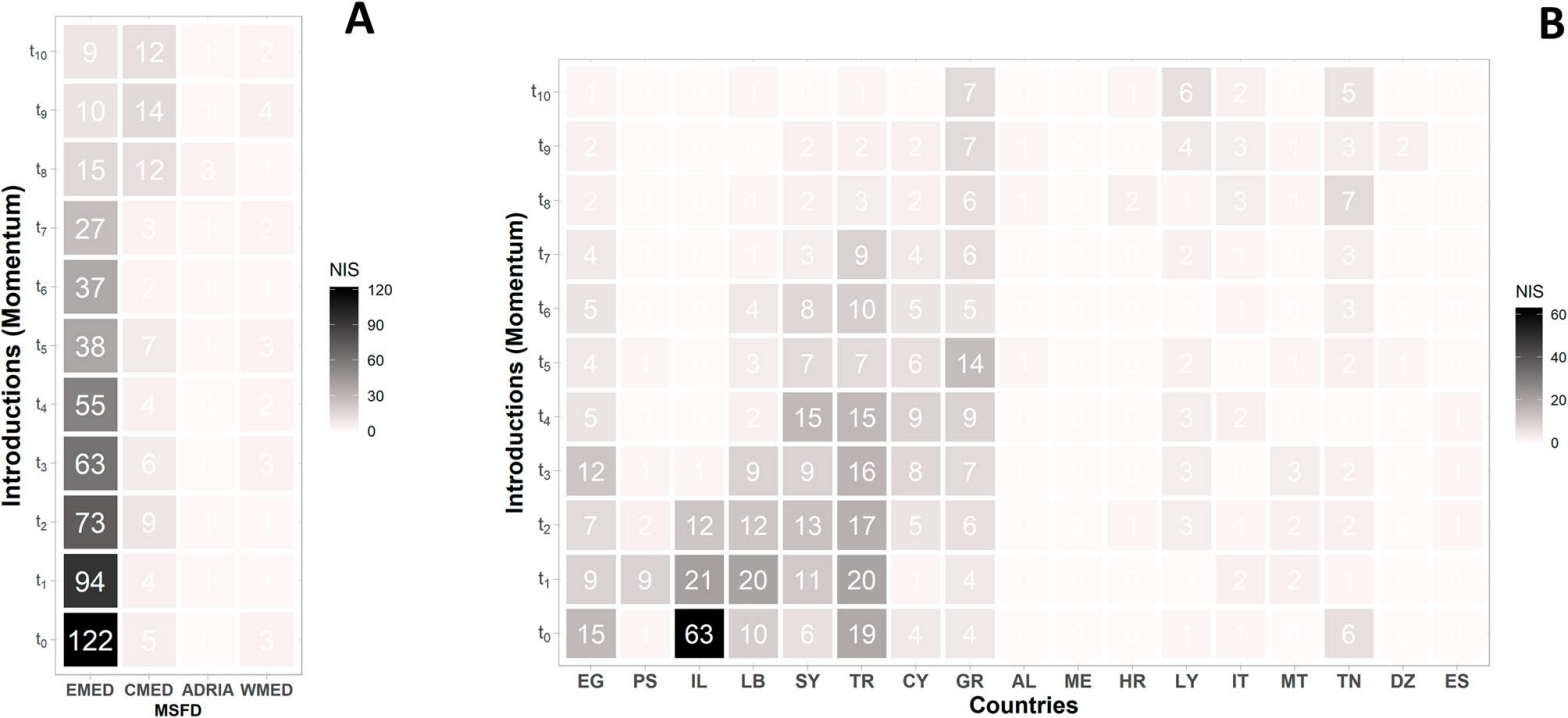
Heatmap representation of introductions based on non-indigenous fishes (NIS) movement (time step; *momentum*) per MSFD region (**A**) and country (**B**). The regions (**A**) and countries (**B**) are ordered with a gradient from east to west, and the abbreviations for each country are displayed in Table 1.

Out of the 130 Indo-Pacific/Red Sea NIS fishes analyzed in our dataset, the vast majority (N = 122; 93.8%) were recorded for the first time in the Eastern Mediterranean basin (i.e., momentum t_0_; Eastern Mediterranean (EMED); Table 1) (Fig. 1A). The total number of records of established species, or the species with at least three successive observations (i.e., t_x_ ≥ t_2_), was estimated as N = 84 and if divided by the total number of NIS (i.e., N = 130), the established species are equal to 64.6% of the NIS fishes in the Mediterranean Sea. By the time of the establishment, 86.9% (N = 73) of NIS fishes were located in the Eastern Mediterranean, 10.7% (N = 9) in the Central Mediterranean (CMED), and 1.2% (N = 1) in the Western Mediterranean (WMED) and the Adriatic Sea (ADRIA), respectively (Fig. 1A).

**Table 1:** List of abbreviations for each Mediterranean country (Country ID) and the respective marine regions based on the Marine Strategy Framework Directive identification (MSFD ID).

Countries	Country ID	Marine regions	MSFD ID
Greece	GR	Eastern Mediterranean Sea	EMED
Turkey	TR		
Syria	SY		
Cyprus	CY		
Lebanon	LB		
Israel	IL		
Palestine Authority	PS		
Egypt	EG		
Libya	LY		
Albania	AL	Adriatic Sea	ADRIA
Montenegro	MT		
Croatia	HR		
Tunisia	TN		
Italy	IT		
Libya	LY	Central Mediterranean Sea	CMED
Italy	IT		
Greece	GR		
Tunisia	TN		
Malta	MT		
Italy	IT	Western Mediterranean	WMED
Tunisia	TN		
Spain	ES		
Algeria	DZ

**Figure 2:**
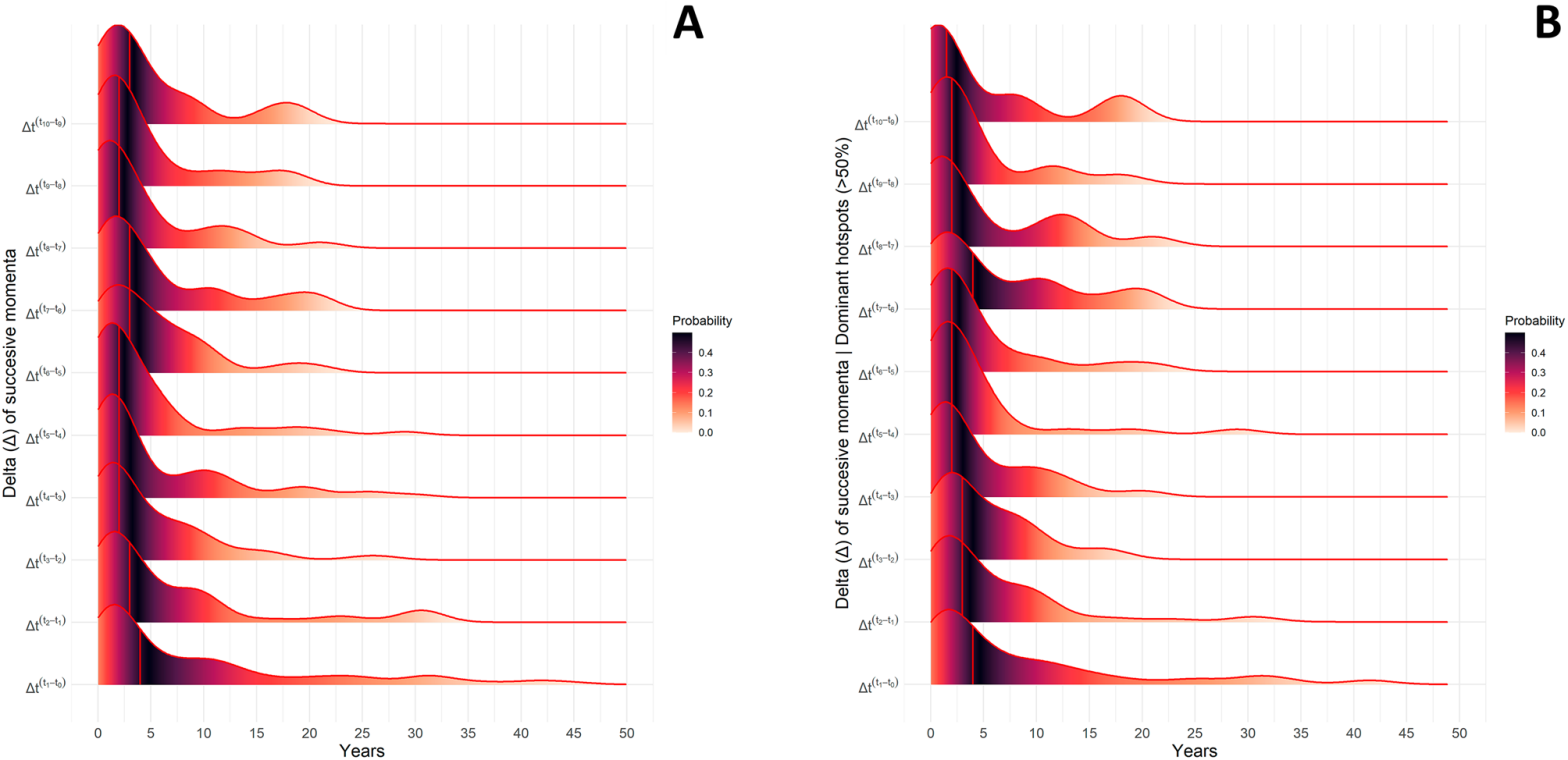
The density curves based on the empirical cumulative density function (eCDF) of the temporal (x; *years*) delta of consecutive sightings or successive momenta (y; *Δ*_*t*_) for all the examined non-indigenous fishes in total (**A**) and the most dominant hotspots of introductions (**B**), defined as the spatial layers which include a percentage greater than 50% of the introductions in total. The red vertical line denotes the median and the scaled color bar (0–0.4 or 0%-40%) the probability of occurrence among values in the eCDF, respectively.

**Figure 3:**
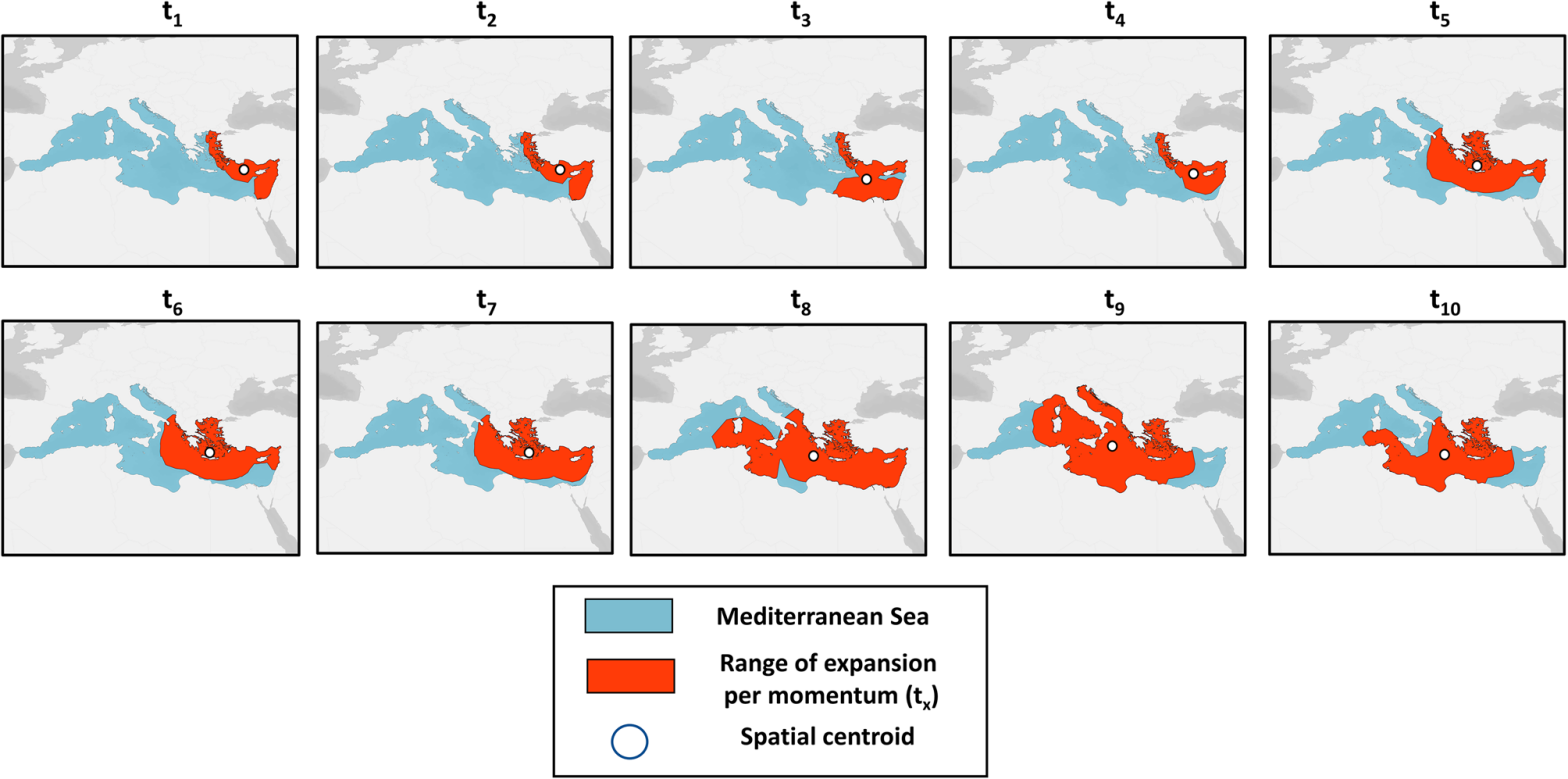
Spatial distribution of the range expansion for the dominant regions (red) of introductions by non-indigenous fishes per analyzed momentum (t_x_) in the Mediterranean Sea (blue). The white circle denotes the spatial centroid of the range polygons for each successive momentum.

Through a higher resolution approach via the national EEZs, the marine coastal waters of Israel showed the highest proportion of introductions (48.5%) at momentum t_0_, with 63 out of 130 NIS fishes being recorded. Apparently, the Levantine Sea demonstrated the entry point of these introductions since Israel, Egypt, Lebanon, Syria and Turkey accounted for 87% of the introductions in total (see Supplementary material; Figs. 1, 2). Furthermore, introductions increase at an accelerating rate through time (Supplementary material; Figs. 1–3). Based on the MSFD classification scheme, the Eastern Mediterranean remained the major marine region of successive introductions from t_0_ to t_7_ (Fig. 1A). It is worth mentioning that after three successive momenta (i.e., t_0_–t_2_), Israel is deposed from being the main area of marine biological introductions, since it exhibited a significant reduction of 63 to 1 NIS (Fig. 1B). On the other hand, the Mediterranean coastal waters of Turkey represented the main receiver of NIS until t_3_ and t_4_, along with Greece which appeared to contribute with increasing NIS introductions events up until t_5_. A key shift point is apparent amidst momentum t_7_ and t_8_ in the Pan-Mediterranean resolution layer, wherein the introductions in the Central Mediterranean (t_7_ = 3, t_8_ = 12) exhibited a discrete prevalence compared to the previously dominant Eastern Mediterranean marine region (t_7_ = 27, t_8_ = 15) (Fig. 1A). The aforementioned momentum shift of introductions towards the Central Mediterranean attains a gradual increasing trend until momentum t_10_ whereas Greece, Libya and Tunisia represent the major introduction hotspots in a descending order (Fig. 1A,B).

Following the numerical analysis on the progressive spread of NIS in the Mediterranean Sea, we estimated the temporal difference (*Δ*; delta) of momenta, or in other words, the sequential timestep among consecutive sightings for all species. Thus, we applied this formula for two different scenarios: (1) all NIS cases (100%), (2) the NIS cases referring to the minimum number of countries with a cumulative proportion greater than 50% of the introductions in total. The temporal delta of each individual case, based on published records, revealed the inferred time required per NIS to spread among consecutive sightings. According to the first (1) scenario, the temporal delta of distribution exhibited that the first momentum (i.e., Δt_1_–t_0_), defined as the transfer from the first towards the second sighting region, was described with the largest range of values from the minimum value at 0 (i.e., transfer from t_0_ to t_1_ within the same year) to the maximum at 65 years. The next momenta displayed gradually narrower time intervals in which the time frame, clearly influenced by the maximum Δt values since minimum ones remained equal to zero, exhibited a reduction of 67% after the sixth delta momentum (i.e., max Δt_7_–t_6_: 21 years), and remained relatively equal until the tenth delta momentum (i.e., max Δt_10_–t_9_: 18 years). All the produced density distributions were characterized as skewed, while the median descriptor (red line) was larger during the first momentum of NIS (median Δt_1_–t_0_: 4 years) and was gradually reduced by 50% after the fourth delta momentum (median Δt_4_–t_3_: 2 years) (Fig. 2A). In other words, the spread of NIS temporally accelerated by 50% after the entry point towards the subsequent momenta. Likewise, a similar pattern was observed in the second scenario (2), in terms of the overall distributional characteristics as well as the median of the empirical cumulative density function (eCDF—see in “Methods”; Fig. 2B), indicating that the subset can be efficiently used in the forthcoming analyses.

**Figure 4:**
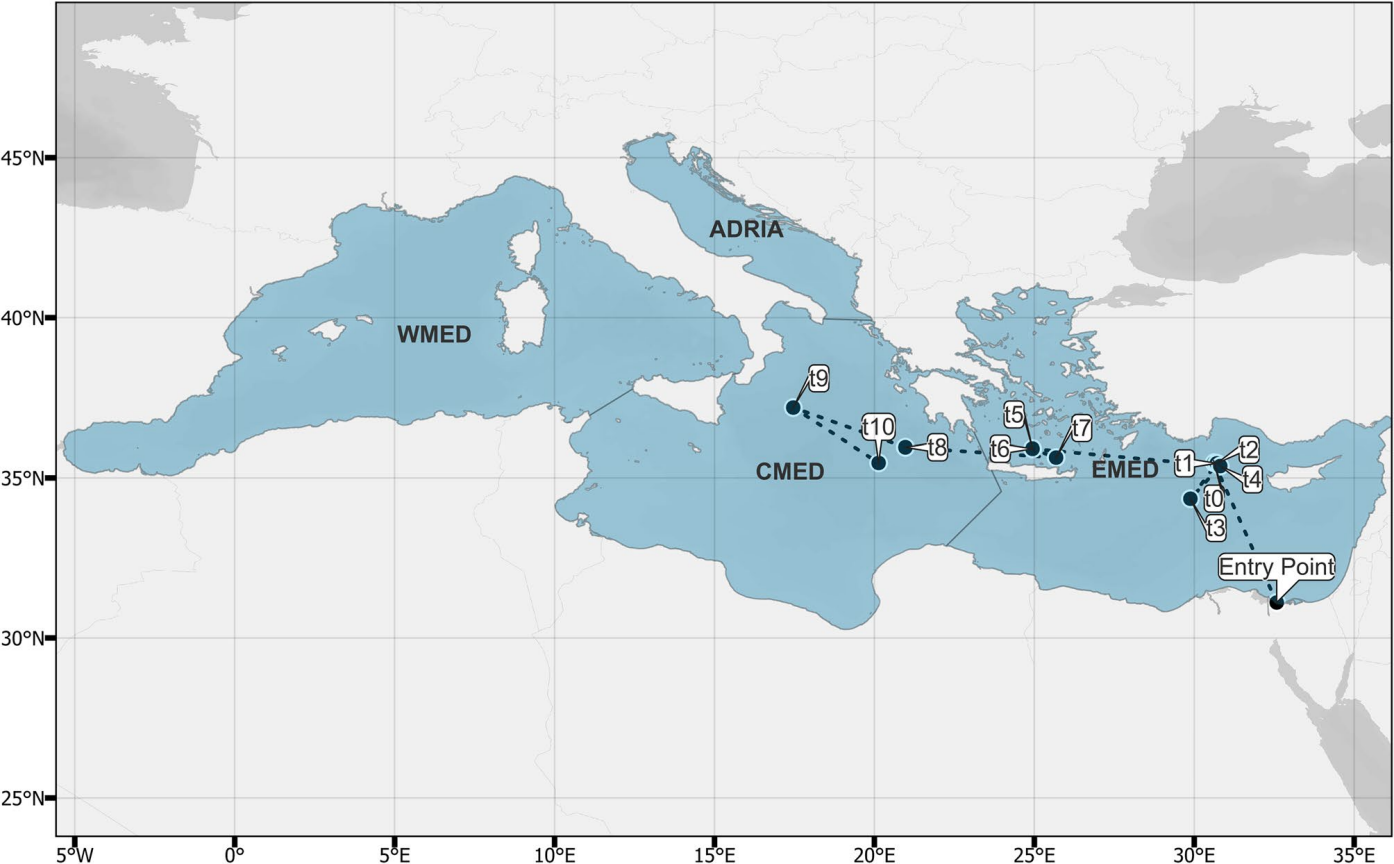
Spatial representation of the progressive movement (*momenta*; black circles: t_x_) of the dominant regions of introductions in the Mediterranean Sea (blue). The progressive trajectory (dotted line) was carried out based on the alignment of the spatial centroids for each successive momentum. The trajectory denotes the connection between the inferred entrance (Suez Canal; Entry Point) to the tenth (t_10_) successive momentum, while the Mediterranean has been classified in the four MSFD regions (EMED; CMED; ADRIA; WMED).

Overall, the numerical analysis of momenta resulted in a distinct successive pattern which allows for the efficient representation of the progressive movement of NIS, covering a large proportion of the Mediterranean Sea (Fig. 1B). To overcome this drawback, we followed the second scenario (2) towards a more efficient spatial representation through EEZ-based polygons along with their respective spatial centroids (Fig. 3). According to this analysis, the Levantine Sea (i.e., Israel, Cyprus, Syria, Turkey) is highlighted as the main region of introductions from t_1_ until t_4_ momenta of NIS movement, while instantly after, Greece is included among the leading countries in the dominant Mediterranean hotspot. Between t_7_ and t_8_, the aforementioned key shift point is apparent, since the dominant hotspot of introductions included for the first time the regions of Central Mediterranean Sea (e.g., southeastern Italy, Tunisia, Western Greece). Following, the Central Mediterranean prevailed as the main region of introductions (t_9_; Fig. 4), although the inclusion of the Adriatic Sea is mainly caused due to the buffer range expansion. At the final momentum of our analysis (i.e., t_10_), the dominant region of introduction is mainly restricted in Greece and the Central Mediterranean coasts of North Africa (i.e., Tunisia, Libya). In order to furthermore simplify the spatial progression of the NIS spread activity, we aligned the spatial centroids of each analyzed momentum in a common trajectory (Fig. 4). Apparently, there is a northward movement of NIS from the inferred entry point (i.e., Suez Canal) to the central regions of Eastern Mediterranean starting from t_0_ until t_4_, respectively. Afterwards, a successive westward trajectory is followed towards the westernmost part of the Eastern Mediterranean (i.e., Rhodes Island, Greece t_5_–t_7_) while in the three final steps of the analysis (i.e., t_8_–t_10_), the momenta are mostly located in the Central Mediterranean.

**Figure 5:**
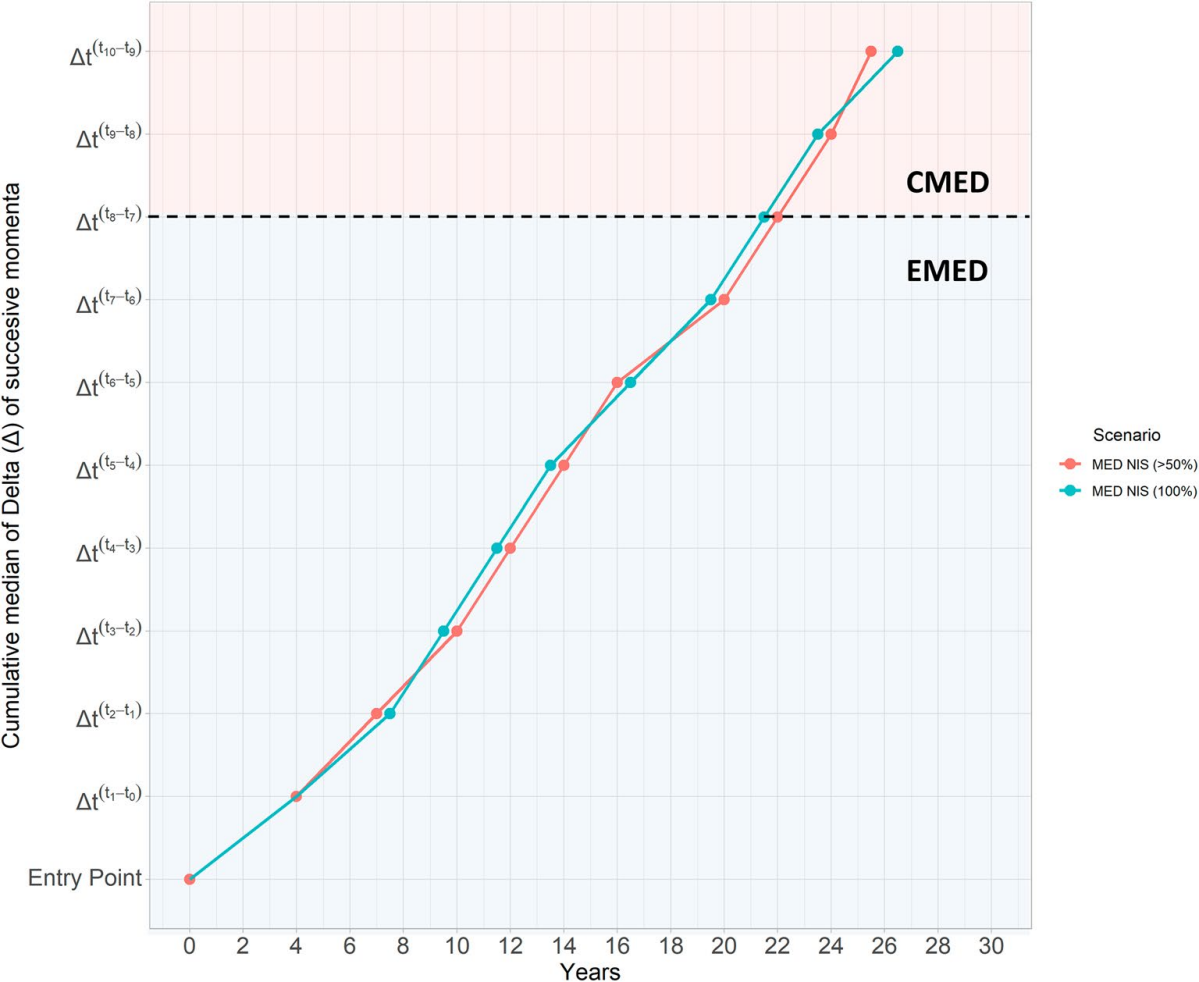
The evolution of the cumulative median of the delta of successive movements (*y; delta of momenta*: *Δt*_*x*+*1*_*−Δt*_*x*_) through time (*x; years*) after the initial introduction for the Mediterranean non-indigenous fishes (MED NIS). The blue and red lines derived from two scenarios: (1) analysis of all MED NIS (100%) and (2) analysis of the dominant MED NIS cases (> 50%). The dotted line expresses the estimated timeframe for a NIS to reach to cross from the Suez Canal (Entry Point) to the EMED (blue), and subsequently towards the CMED (red) at 21.5–22 years for both scenarios.

Based on the cumulative delta median of successive movements, we investigated the time intervals between steps of introduction, or momenta. By assuming that the entrance of a species in the Mediterranean occurs at t = 0, the initial movement of a NIS from t_0_ to t_1_ had a duration of 4 years for both scenarios (1) and (2), respectively (Δt_1_–t_0_; Fig. 5). Following, the successive momenta exhibited relatively equal time intervals with an average of 2.4 and 2.31 years for scenario (1) and (2), respectively, with both scenarios exhibiting a common cumulative increasing trend. As a matter of fact, the deviation of each momentum between the two scenarios ranged from 0% (minimum; Δt_1_–t_0_) to 6.6% (maximum; Δt_2_–t_1_). In conjunction with the dominant trajectory (Fig. 4), it appears that the overall time needed for a NIS, to reach the Central Mediterranean from its entry point in the Suez Canal, is estimated between 21.5 and 22 years for scenario (1) and (2), respectively (Fig. 5).

**Figure 6:**
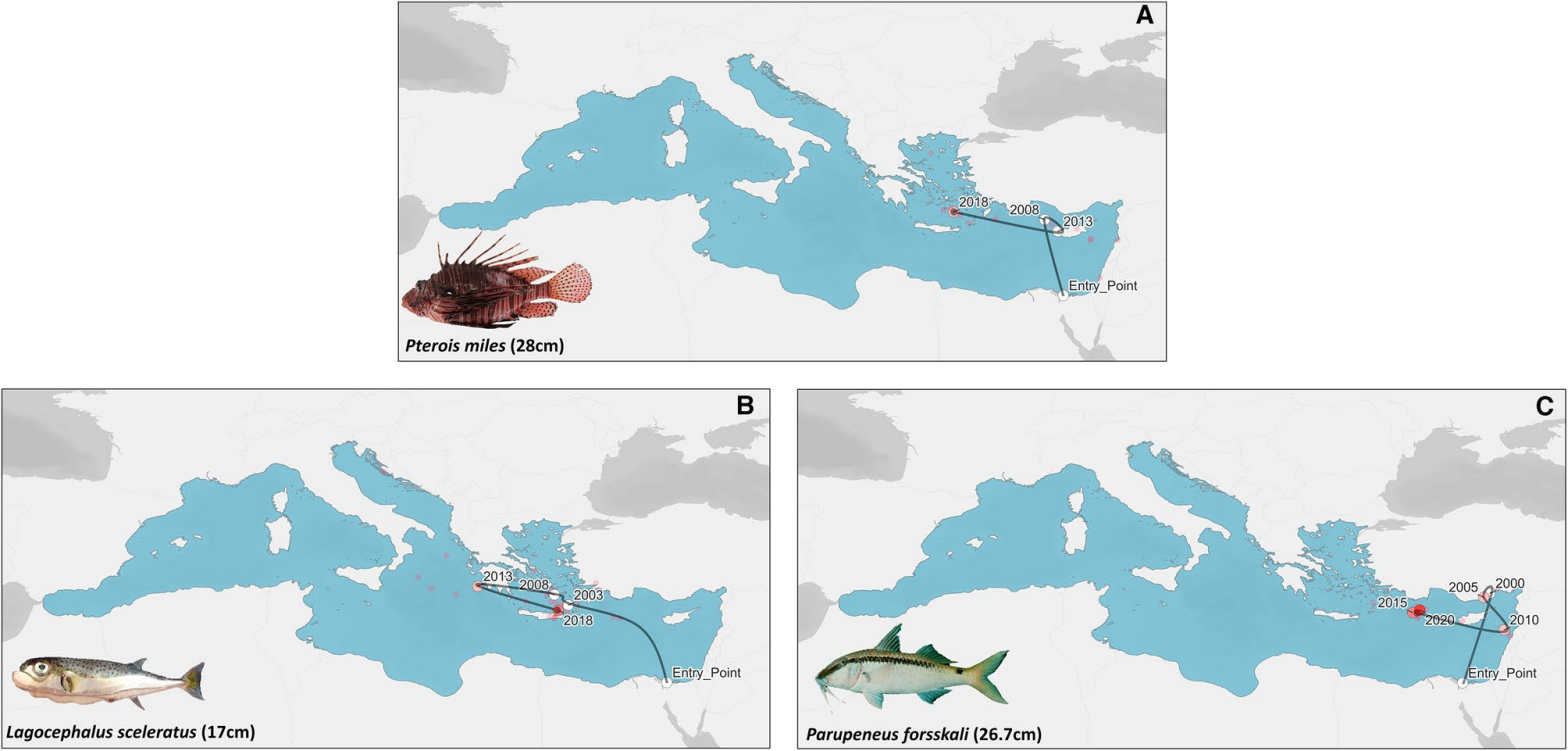
Spatial representation of the five-year average centroids (large circles) of three emblematic cases of invasive alien species in the Mediterranean Sea (*P. miles* **A**; *L. sceleratus* **B**; *P. forsskali* **C**). The black line demonstrates the route from the Entry Point (Suez Canal) to the endmost 5-year moving average. The transparent layer of centroids (small circles) exhibits the annual average centroids of each species records.

For the needs of the momentum approach validation, we compared our results with the actual invasion history of three emblematic invasive alien species (IAS) fishes of the Mediterranean (i.e., *Pterois miles* Bennet (1828), *Lagocephalus sceleratus* (Gmelin, 1789)*, Parupeneus forsskali* (Fourmanoir & Guézé, 1976)). Regarding the spatial dimension of their expansion, all species validated the north-westward trajectory, as it was priorly produced through the momentum approach (Fig. 6). In the case of *P. miles*, its invasive activity has an actual duration of 15 years (i.e., 2008–2022), with the centroid of its endmost location being positioned in the South Aegean Sea (Fig. 6A). Hence, based on the momentum approach (Fig. 4), the actual endmost location of *P. miles* is adjacent to the centroid of t_5,_ which corresponds to an inferred duration of invasive activity at 13.5 years (i.e., Δt_5_–t_4_; Fig. 6). Thus, the temporal validation resulted in a 90% agreement (Table 2). Likewise, the *L. sceleratus* and *P. forsskali* were characterized with an actual duration of invasiveness at 17 years (i.e., 2003–2019) and 19 years (i.e., 2000–2018) and their final spatial centroids overlapped with t_7_ and t_5_ momenta, respectively. Accordingly, the inferred duration of the invasive activity for *L. sceleratus* and *P. forsskali* resulted in 19.5 and 13.5 years (i.e., Δt_7_–t_6_; Δt_5_–t_4_; Fig. 6), respectively, thus achieving a validation of 87.2% and 71%, respectively (Table 2).

**Table 2:** Validation of the momentum approach based on three invasive alien species (IAS) according to their trajectory and temporal movement in the Mediterranean Sea as it is depicted in Fig. 6.

Lessepsian species	Actual duration of invasive activity (years)	Inferred endmost location based on momentum analysis (Fig. 4)	Inferred duration (years) of invasive activity based on momentum analysis (Fig. 5)	Temporal validation (%)	Trajectory validation
*Pterois miles*	15*	t^5^	13.5	90	Validated
*Lagocephalus sceleratus*	17	t^7^	19.5	87.2	Validated
*Parupeneus forsskali*	19	t^5^	13.5	71	Validated

*First observation back in 1991, most probably a non-successful invasion^53^, although invasive activity started after 2008.

## Discussion

The Mediterranean Sea is considered as a marine biodiversity hotspot since it hosts approximately 17,000 marine species, 20.2% of them being endemic, with the endemic fishes being estimated at 12%^5^. The last five decades marine scientists started to intensively explore the phenomenon of marine biological invasions that imperil marine ecosystems. Several pioneer study efforts have investigated non-indigenous species’ patterns, pathways and impacts in the Mediterranean Sea and its adjacent regions^6,30,31^. In parallel with theoretical and baseline knowledge, the nature of historical spatio-temporal patterns of NIS introductions is equally significant to assess their spread^32^ and temporal variability^28^, by also contributing to biogeographic and macroecological research. Given the importance of the time component, especially in terms of the dynamic momenta of movement, we provide a novel analysis framework regarding NIS detection records as a benchmark approach for marine invasions in the Mediterranean Sea.

One of the most interesting results presented herein is that the majority of NIS fishes require 4 years approximately to spread from the first towards the second region of detection (i.e., Δt_1_–t_0_), and 21.5–22 years in total (i.e., Δt_8_–t_7_), to further spread westwards from the Eastern to the Central Mediterranean. The northward trajectory depicted for the scenario (2) is validated through several other works in the Mediterranean and the Atlantic Sea as a poleward movement induced by environmental changes^27,33^. The underlying mechanism is attributed to temperature levels, since the distribution of ectotherm marine species, as the vast majority of fishes^34^, is a physiological response to sea temperature, being also delimited by their realized thermal limits^35^. Another important finding is the existence of a westward tipping point in the southern Aegean; this trajectory continues until the final momentum of our analysis, being positioned between western Greek and Libyan coasts. We have observed that the majority of NIS records are significantly reduced towards the Central Mediterranean, a finding in agreement with previous studies showing a significant decrease of introductions in the North Aegean Sea and the Straits of Sicily^28^.

Although meta-analysis studies of historical introductions have demonstrated a consistent northward course, aligning with the initial momenta of our analysis, bioclimatic projections indicate that climate change will force marine species to shift further eastwards and northwards, that is to say towards the Aegean and Adriatic Seas^36^. These geographic range extensions or contractions as a consequence of warming may facilitate a “*cul-de-sac*”, or a final terminal, hence increasing the risk of species extinction^37^. Regarding the response of NIS to their new habitats, the “*vacant*” or “*empty niche*” hypothesis was proposed for the Lessepsian migration in the Eastern Mediterranean, based on the assumption that regions that are characterized by low diversity host native species unable to efficiently exploit the provided resources, facilitate suitable conditions for establishment to newcomers^38^. Admittedly, this is the case for the Levantine Sea, a region with low endemism of fishes^5^, where during 1990–2011 NIS fishes established and replaced native fish of similar ecological position, also leading to their biomass and abundance decline^12^. Concerning species’ establishments in the Mediterranean Sea, the “*tens rule*” hypothesis (i.e., 10% of introductions become established) has long since been disproven across several NIS taxa^9^. Likewise, our results indicated that 64.6% of NIS fishes are already established, while the overall time needed for a species to be established is approximately 10 years (i.e., Δt_3_–t_2_: scenario (1) = 9.5 years—scenario (2) = 10 years).

In order to verify our methodological framework, we utilized the actual geocoordinates of three widely expanded IAS in the Mediterranean (namely *P. miles*, *L. sceleratus*, *P. forsskali*). Additional validating support to our work is derived through the recently published least-cost distance path for *P. miles*, using its occurrences and sea current velocities^39^, which is almost identical to the north-westward trajectory of NIS presented as an output of the momentum analysis. With regards to the case of *P. forsskali*, its rapid spread reveals a similar pattern with that of *P. miles* and *L. sceleratus*. Undoubtedly, the most notorious and widespread among the three species used for the validation of our results is *L. sceleratus*. Even though our results exhibit a north-westward trajectory in the Mediterranean Sea, a recent published work forecasted that it may enter and establish the Black Sea through the Dardanelle Strait^24^. In fact, *L. sceleratus* has been documented twice, both in the Sea of Marmara (i.e., connection point between Mediterranean-Black Sea) and the Black Sea^40^. If this speculation is confirmed, the Black Sea ichthyofauna may be in peril considering its high degree of endemism, estimated almost three times higher than in the Mediterranean (i.e., 31.22%)^41^. In the same context, a similar work of habitat niche modelling was carried out for *P. miles* and stated that the Adriatic and Alboran Seas constitute unfavorable habitats for the spread and invasion of lionfish^39^. Unfortunately, the aforementioned statement was disproved, as the species was sighted in both regions less than 3 years after the publication^42,43^. Therefore, it is imperative that the development and implementation of predictive distributional models should always be under continuous and rigorous re-evaluation.

As it has been mentioned before, existing literature enhance the validity to our results, although we identify limitations towards future relevant studies. Initially, the present simplified framework did not take into account environmental variability nor multispecies interaction. We also agnostically analyzed given data of NIS movement based on published records, by assuming that their spread through time is the product of complex biological and environmental susceptibility^44^. With regards to the spatial layer of the presented analysis, we proceeded in the selection of large marine regions (i.e., EEZs, MSFDs). This selection occurred due to the representation of EEZs and MSFDs as substantial administrative units which therefore can be used towards effective management initiatives. It should be stated that the selection of continental shelf zones, as regions inhabited by the majority of NIS, would have plausibly led to a spatial pattern more adjacent to coastal areas. However, the variety of the Mediterranean topography (i.e., discontinuity of continental shelves) and the pathways of NIS would have resulted into a more complex and potentially incomprehensible invading pattern. Nevertheless, additional research focus should be given by future studies on implementing various spatial layers (e.g., FAO fishing areas and divisions, marine ecoregions, continental shelves) to enhance the presented results by incorporating the proposed *momentum* framework. In the same context, certain bias may occur due to the lag in detection of NIS records which varies through space and time^15,28^, although it has been significantly reduced after the ‘80s due to the increasing scientific efforts at a regional scale. Despite the fact that sampling efforts have been increased through time, there are still several data-scarce regions in the Eastern Mediterranean Sea. By taking this into consideration it is easily accepted that large-scale research studies which combine cross-validated scientific and citizen-science based information, significantly reduce such biases and clarify the produced patterns. A recent Pan-Mediterranean survey on the local ecological knowledge of fishers confirmed the north-westward expansion of NIS, despite the uneven research efforts that has been historically observed in the region^45^. Finally, certain species may be potentially described with a non-northward successive movement (e.g., Adriatic Sea: t_0_, Gulf of Tunis: t_5_; Levantine Sea: t_8_), although our results provide robust evidence into validating the initial hypothesis that a common trajectory exists and that the implementation of large datasets mitigates such obstacles both under a quantitative and qualitative aspect.

Notwithstanding that this work provides some exploratory analysis results, numerous opportunities can arise by integrating several interdisciplinary approaches. Through our perspective, an urgent utilization of the produced spatial and temporal delineated trajectory into marine spatial planning is imperative, a field wherein the spread of invasive species has been largely neglected^46^. An additional consideration should be given to the integration of momentum time intervals and the relationship between species’ biological traits and environmental conditions especially under the context of global changes. Specifically, a major focus should be given to temperature tolerance and extreme aperiodic fluctuations which have been proved to considerably affect the spread, distribution and population status of invasive^47^ and native species^48^. Moreover, the questionable effect of Marine Protected Areas (MPAs) on the spread of NIS could be further examined using the successive spatio-temporal spread of NIS, since the dynamic evolution of the three stages of arrival, establishment and spreading would provide additional support either on the “*biotic resistance*” or “*biotic acceptance*” hypotheses^46^. Besides considering MPAs as refugia for native species against marine invasions under climate change^27^, the effect of shallow versus deep marine habitats should be also explored since the rate at which marine species need to spread in order to respond to climate-induced habitat shifts is 30 times higher in the sea surface compared to the sea bed^32^. A critical view on previous management initiatives facilitates a productive discussion on whether marine invasions should be examined under the light of their ecological role and their effect on ecosystem functioning^46^ or through a militaristic-eradicative, potentially unsustainable, management strategy that has been traditionally implemented^44^.

Marine ecosystems are facing constant changes due to natural and, most importantly, human related processes. The Mediterranean Sea is perceived as one of the most impacted marine areas, considering the enclosed nature of the basin and the historical presence of intensive human activities. Despite the application of large-scale distribution models, limited attention has been paid to the dynamic patterns of successive movements of NIS across the region. Through an open-source standardized methodological framework, we concluded that NIS accelerate their movement after their initial entry in the Levantine Sea by advancing their spread almost every 2.5 years, while they require approximately 22 years to reach the Central Mediterranean basin. Additionally, a discrete north-westward trajectory is revealed among NIS. The presented outcomes would be enriched through the integration of concepts such as environmental variability, biological traits, intra- and interspecific interactions and dynamic trends of NIS.

## Methods

### Dataset

Based on an extensive scientific literature survey conducted by the Hellenic Centre for Marine Research (HCMR), we have constructed the largest dataset so far regarding information of the year and the country of observation for a total of 130 NIS of Indo-Pacific/Red Sea origin that have been introduced in the Mediterranean Sea by June 2023, most likely spreading via the Suez Canal either unaided (pathway corridor) or with vessels (Pathway Transport-Stowaway). Specifically, the dataset contained all NIS detection records through time and space. The main sources of information were retrieved from records included in the georeferenced HCMR offline database hosted by the European Alien Species Information Network^49^ and from the Occurrence Records of Mediterranean Exotic Fishes database^11^. Additionally, data from peer-reviewed published sources and scientifically validated grey literature were included. All records have been thoroughly examined and pre-processed^50^, hence, false taxonomic identifications, duplicated occurrences and unreliable spatio-temporal data have been omitted.

### Data numerical analysis

The final dataset contained the spatial and temporal records of NIS observations starting from the initial towards consecutive sightings for all the species. We define the event of sighting as momentum (i.e., momentum of first record; t_0_) in order to enable the progressive analysis of the subsequent successive steps in an ascending temporal order (i.e., t_1_, t_2,_ t_3_… t_x_). Through this approach we were able to simplify and, at the same time, group the analysis per individual NIS. As a next step, we assigned boolean attributes to the successive momenta to allocate presence or absence of NIS in each EEZ and MSFD subregions of the Mediterranean Sea (i.e., Exclusive Economic Zones-EEZs, Marine Strategy Framework Directive-MSFD; Table 1). We excluded Mediterranean countries which lack records of NIS introductions from subsequent analyses.

The momentum approach allows for an efficient interpretation of NIS trajectories, however, if examined on the species level, the progressive trajectory of NIS is incomprehensible. In addition, sophisticated comparisons cannot be implemented on the species-level variations since the spatial scale of analysis may hinder the emergence of patterns of NIS introductions. To this end, records of each momentum were aggregated for NIS and heatmaps were produced to visualize the phenomenon of NIS introductions in the Mediterranean Sea. We considered species with at least three successive observations (i.e., t_x_ ≥ t_2_) as established NIS^15,25^. Hence, with the use of the temporal delta difference of momenta (*t*_*x*_) for each NIS (*i*), we calculated the temporal change of their movement (i.e., Δ_t[i]_ = Δt_x+1[i]_ − Δt_x[i]_). Afterwards, we demonstrated the distribution of each Δ_t[i]_ of all the NIS cases through time (years), by employing the empirical cumulative density function (eCDF)^51^. In simpler terms, by utilizing a simple distributional representation of each Δ_t[i]_ we visualized for the first time the changes and the related probabilities of the time required for all NIS to proceed to consecutive sightings.

### Spatial analysis

Following the numerical analysis, we proceeded with the visualization of the successive trajectories of NIS momenta in space. To do so, we imported EEZs retrieved by the Marine Regions geodatabase^52^ and MSFD Mediterranean subregions layers into the open-access environment of QGIS. Due to the large number of records for the 130 NIS (N = 772), it was expected that at each momentum the presence of NIS would be assigned to multiple marine regions; thus, overlapping would lead to an uninterpretable spatial pattern. In order to overcome this obstacle, we investigated two different scenarios of spatio-temporal analysis: (1) for all observation cases (100%), (2) for the minimum number of countries that their observation cases constitute at least 50% of the detection records, defined as the dominant Mediterranean hotspots of introductions (> 50%). According to the second scenario, a buffer zone with 1° (min) to 3° (max) degrees of expansion was applied in order to encompass polygons and to provide additional spatial expansion that could favor the recorded NIS. This spatial resolution framework allowed to increase significantly the level of spatial display; however, in order to unravel the trajectory of NIS introductions an additional display was applied. We estimated the geocoordinates of the spatial centroid of each momentum-based buffered polygon, while afterwards the centroids have been aligned, starting from the inferred entry point (i.e., Suez Canal) towards the first record and, ultimately, to the successive momenta (i.e., t_0_–t_10_). Likewise, for the second scenario, we estimated the temporal delta difference distribution for each Δ_t[i]_ for the NIS cases belonging only to the dominant Mediterranean hotspots. Since the density curves of the examined Δ_t[i]_ of both scenarios (1) and (2) resulted in skewed (i.e., non-gaussian) distributions, we applied the median descriptor to quantify the progressive succession among momenta. Afterwards, we proceeded in the calculation of the cumulative temporal delta of the successive momenta by using the eCDF’s median of each Δ_t[i]_ as a proxy, in order to approximately estimate the overall time needed for a NIS to expand its spread into successive Mediterranean regions starting from the Suez Canal. Finally, we examined the relationship between the successive delta intervals and the subsequent cumulative trend for both scenarios (1) and (2). The potential difference of these two approaches was estimated by calculating the percentage deviation between the temporal delta of each scenario for each momentum.

### Validation/ground truth procedure

Even though the aforementioned methodology utilizes published detection records and implements a standardized numerical analysis framework, the patterns produced should be validated through species-specific invasion cases in order to justify the method’s applicability and repeatability. As a result, we selected three emblematic and well-known invasive alien species (IAS; i.e., *P. miles*, *L. sceleratus, P. forsskali*) that exhibit a well-studied spatio-temporal expansion in the Mediterranean Sea. It should be noted that we excluded the first literature observation of *P. miles* from the Levantine Sea in 1991, since it was most probably a non-successful invasion attempt of a single individual^53^. We analyzed the species’ movement in order to examine whether their spread dynamics correspond to our produced patterns both in space and time. At a first stage, we used the exact record coordinates of the three IAS instead of the qualitative EEZ-based data, in order to strengthen the produced results. Since for each selected IAS, multiple records existed within a year, we estimated their annual- and 5-year average spatial centroids. The validation took place by investigating the species’ trajectory and their actual spread duration (i.e., Δt_x_ − t_x-1_; x: latest momentum), compared to the results obtained (Fig. 4). Finally, temporal information related to the inferred spread/invasion activity was retrieved through scenario (1) of the momentum approach in order to include all analyzed NIS.

### Supplementary Information

Supplementary Information.

## Data Availability

Data used for this study, the produced figure outputs and the open-source code script (R programming language v4.3.2) are publicly available and can downloaded through the project’s dedicated GitHub repository (https://github.com/Vagenas7119/DLSMed). For any type of additional inquires contact with the corresponding author.
