# Supplementary material for: Decoding the spread of non-indigenous fishes in the Mediterranean Sea
Source: Sci Rep. 2024 Mar 20;14:6669. doi: 10.1038/s41598-024-57109-8 (PMC10954742; doi:10.1038/s41598-024-57109-8)
Supplement: Supplementary file 1 — Supplementary Information. [file 41598_2024_57109_MOESM1_ESM.pdf]

Supplementary Material

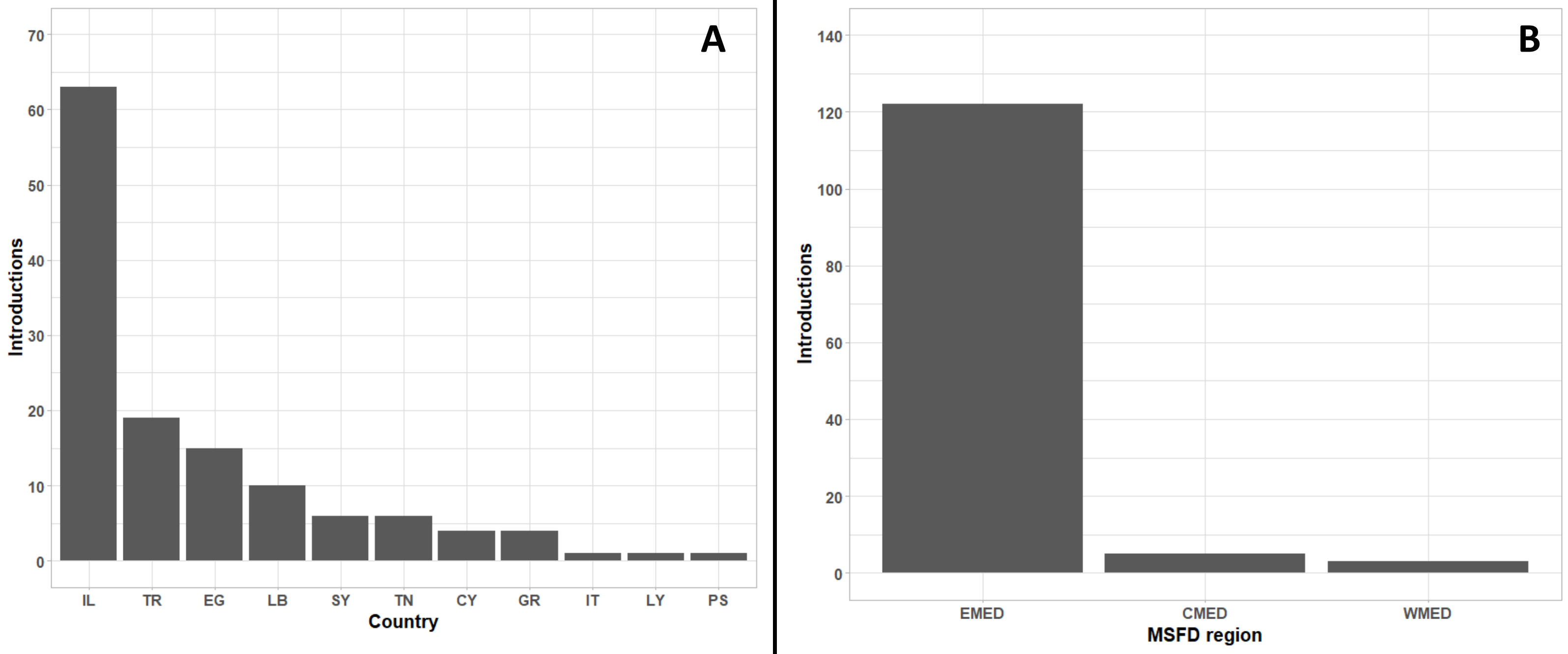

[Sup_Fig1] Figure 1. The allocation of first sighting introductions (i.e., t_0) of non-indigenous fishes (NIS) per country (A) and MSFD regions (B). The abbreviations of each country and MSDF regions can be found in Table 1.

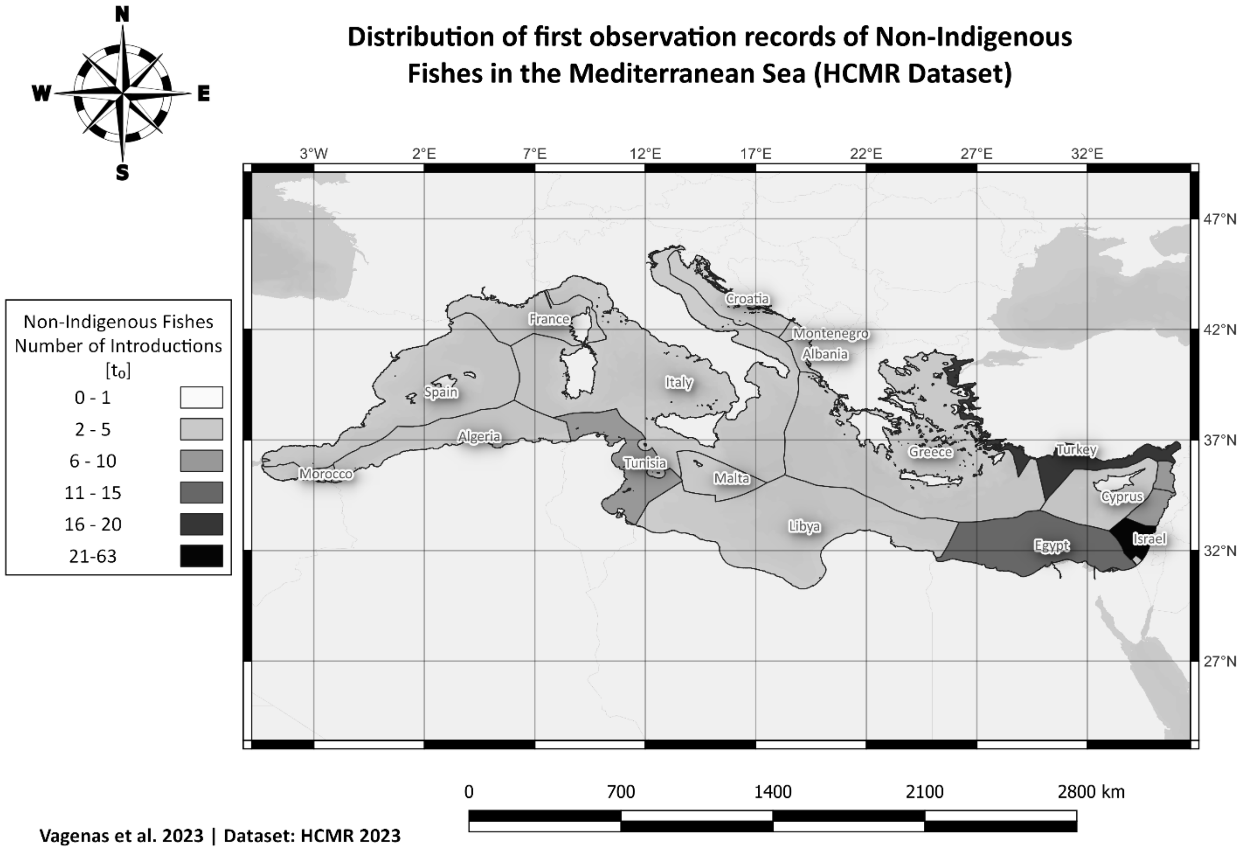

[Sup_Fig2] Figure 2. The spatial distribution of first records (t_0) of non-indigenous fishes in the Mediterranean Sea up to 2023. The spatial polygons represent the Exclusive Economic Zones (EEZs) of each Mediterranean country.

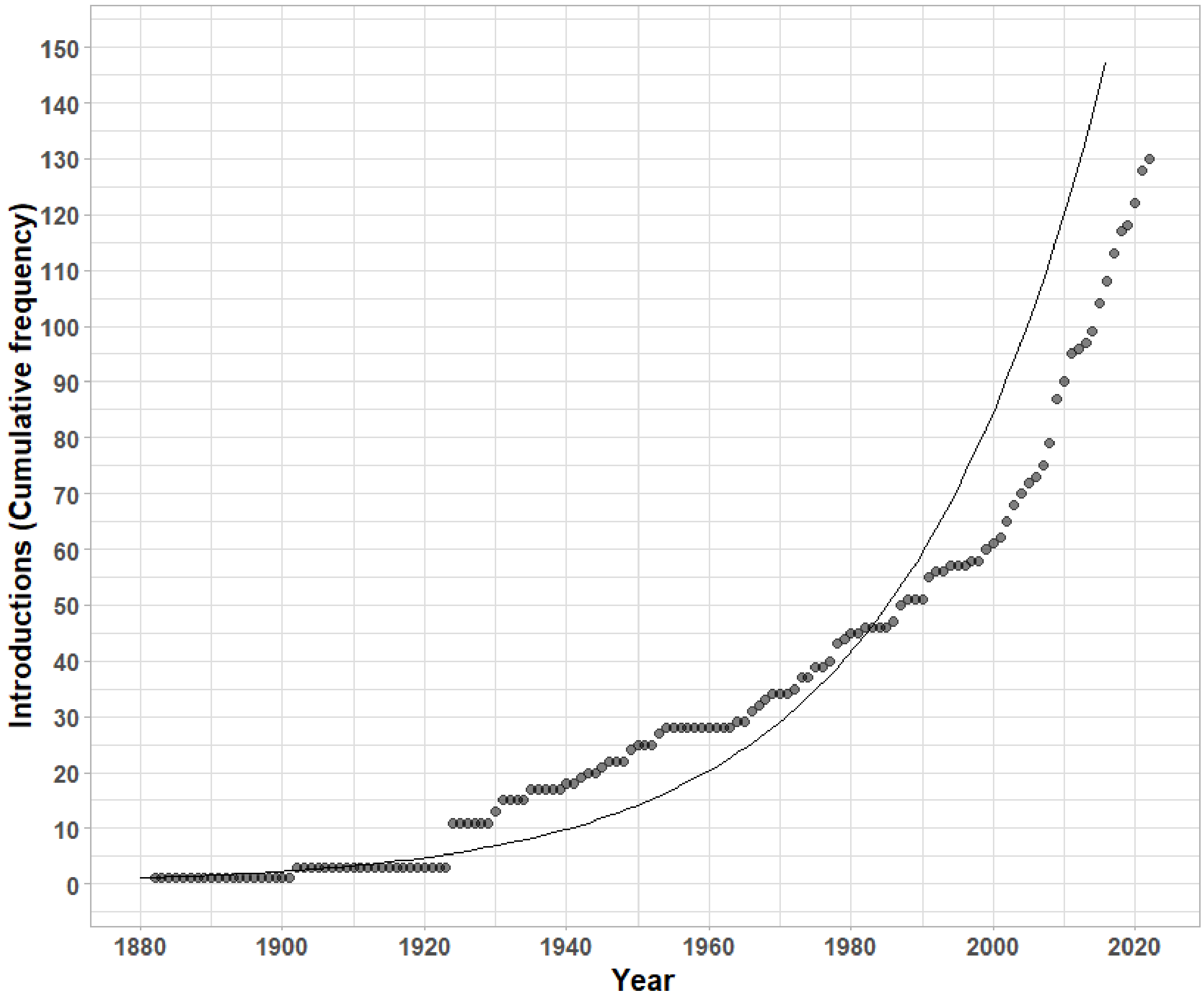

[Sup_Fig3] Figure 3. The cumulative frequency of non-indigenous fishes introductions in the Mediterranean Sea from 1882 to 2023.
